# Thermal Behavior of Curaua-Aramid Hybrid Laminated Composites for Ballistic Helmet

**DOI:** 10.3390/polym15153214

**Published:** 2023-07-28

**Authors:** Natalin Michele Meliande, Michelle Souza Oliveira, Maurício Ferrapontoff Lemos, Artur Camposo Pereira, André Ben-Hur da Silva Figueiredo, Sergio Neves Monteiro, Lucio Fabio Cassiano Nascimento

**Affiliations:** 1 Department of Materials Science, Military Institute of Engineering—IME, Praça General Tibúrcio, 80, Urca, Rio de Janeiro 22290-270, Brazil; nmeliande@gmail.com (N.M.M.); camposo@ime.eb.br (A.C.P.); abenhur@ime.eb.br (A.B.-H.d.S.F.); snevesmonteiro@gmail.com (S.N.M.); lucio@ime.eb.br (L.F.C.N.); 2 Modeling, Metrology, Simulation and Additive Manufacture Section, Brazilian Army Technology Center—CTEx, Avenida das Américas, 28.705, Guaratiba, Rio de Janeiro 23020-470, Brazil; 3 Group of Materials Technology, Brazilian Navy Research Institute (IPqM), Rio de Janeiro 21931-095, Brazil; engmlemos@gmail.com

**Keywords:** natural fiber, aramid, curaua non-woven mat, hybrid composite, ballistic helmet, thermogravimetric analysis, thermogravimetric derivative, differential thermal analysis, thermomechanical analysis

## Abstract

Hybrid composites are expanding applications in cutting-edge technology industries, which need materials capable of meeting combined properties in order to guarantee high performance and cost-effectiveness. This original article aimed for the first time to investigate the hybrid laminated composite thermal behavior, made of two types of fibers: synthetic Twaron^®^ fabric and natural curaua non-woven mat, reinforcing epoxy matrix. The composite processing was based on the ballistic helmets methodology from the North American Personal Armor System for Ground Troops, currently used by the Brazilian Army, aiming at reduced costs, total weight, and environmental impact associated with the material without compromising ballistic performance. Thermal properties of plain epoxy, aramid fabric, and curaua mat were evaluated, as well as the other five configurations of hybrid laminated composites. These properties were compared using thermogravimetric analysis (TGA) with its derivative (DTG), differential thermal analysis (DTA), and thermomechanical analysis (TMA). The results showed that the plain epoxy begins thermal degradation at 208 °C while the curaua mat at 231 °C and the aramid fabric at 477 °C. The hybrid laminated composites curves showed two or three inflections in terms of mass loss. The only sample that underwent thermal expansion was the five-aramid and three-curaua layers composite. In the third analyzed temperature interval, related to the glass transition temperature of the composites, there was, in general, an increasing thermal stability behavior.

## 1. Introduction

With the advance of cutting-edge industry, there is a need for composites, with properties that are not possible to obtain using only one type of filler material. Thus, hybrid composites emerged, bringing the combination of different filler materials that generate better properties than each material separately [1]. Hybridization proved to be a very efficient way to improve the properties of composites and, consequently, the study using different types of materials as reinforcement has become common in recent times [2,3,4,5]. Materials such as glass fiber [6], carbon fiber [7,8], industrial waste [9] and even natural fibers [10,11] have gained prominence for these applications.

Hybrid composite materials are being applied in many instances, with a wide range of temperature variations. In this respect, it is very important to study their thermal behavior once that large temperature differences can affect the structural stability of hybrid composites [12]. Their thermal properties are influenced both by the nature and properties of the polymer and the reinforcement fillers [6]. The occurrence of dimensional variation due to thermal stress is undesirable. Very high thermal stress can lead to deformations and impair the adhesion of the laminated layers, resulting in a decrease in mechanical performance. As a consequence, it is recommended to reinforce hybrid composites with smooth sheets or weave fabrics due to their high structural stability [6,12,13]. Another fact present in the industry today is the sustainable concern associated with how to produce more with less waste and reduce environmental impacts [14]. Deforestation through human activities and global warming has been a significant challenge for materials scientists [14]. Consequently, natural fiber reinforced composites have been increasingly used in several technological sectors as exemplified by the diversified application of curaua fiber (Ananas erectifolius) incorporated into polymer matrix for civil construction [15,16], automotive [17], aeronautic [1] and defense [18,19,20].

Curaua is a plant native from the Brazilian Amazon, of the same family of pineapple, whose fibers are applied by different industries for partial replacement of fiberglass [21,22,23]. This species can be found in other neighboring countries, such as Guyana, Colombia, Venezuela, and Suriname, under different names [24], such as askurowa, curaua, caroa, wild pineapple, and others. Studies and research carried out in Brazil and abroad proved that curaua fibers present excellent mechanical results, demonstrating resistance comparable to glass fibers. Because of this, curaua fibers gained prominence among research groups with a socio-environmental perspective [25].

In parallel, the synthetic aramid fiber exhibits an exceptional combination of high modulus and high tensile strength, high toughness, high impact strength, and high resistance to creep and fatigue failure [26,27]. In addition, these fibers are resistant to combustion and stable over a wide temperature range [28,29]. Aramid fibers are susceptible to degradation only by strong acids, bases, and ultraviolet radiation [30,31]. As aramid fibers are flexible and ductile, they can be processed by textile operations. On that account, aramid fabrics with different yarns, weaves, and weights display different mechanical properties [32,33]. The aramid family includes commercial fabrics called Kevlar^®^, Nomex^®^, Technora^®^, Twaron^®^, among others. Para-aramid fibers, such as Kevlar^®^ and Twaron^®^, are slightly different with excellent strength-to-weight ratio and high tenacity [34]. Composites made of high-density polyethylene (HDPE) reinforced with Twaron^®^ fibers show a significant increase in mechanical and thermal properties [35,36]. The Twaron^®^ fiber, produced by Akzo™ (The Netherlands), has a similar chemical structure to the Kevlar^®^ fiber, produced by Du Pont™ (USA) [37]. Results show that para-aramids have similar thermal stability, but their thermal degradation temperatures and activation energies in the air are different [38]. Another point is that to obtain maximum performance from a composite, a strong fiber with good matrix compatibility is required [6,37]. With regard to thermal and mechanical properties, Twaron^®^ fibers are good candidates as reinforcement [36,37].

This original article presents for the first time an investigation of the thermal behavior of hybrid composites made of two types of fibers, the synthetic one, the Twaron^®^ fabric, and the natural one, the curaua non-woven mat, both incorporated into polymeric epoxy matrix. The production of US ballistic helmets, currently used by the Brazilian Army, was considered, with the aim of reducing costs and environmental impact associated with the material, without compromising ballistic and thermal performance [39,40]. Thermogravimetric (TGA) and thermomechanical (TMA) analyses obtained the relationship between property and temperature. There was no significant variation in the analyzed temperatures, which proves that the hybridization made with natural curaua mat and synthetic aramid fabric has great potential in the manufacture of ballistic helmets.

## 2. Materials And Methods

### 2.1. Materials and Composites Processing

For the polymer matrix of the composites, a commercial epoxy resin of the diglycidyl ether type of bisphenol A (DGEBA) was used, hardened with triethylene tetramine (TETA), in the proportion of 13 parts of hardener to 100 parts of resin, both produced by Epoxyfiber (Rio de Janeiro, Brazil). To reinforce the composite materials proposed in this study, aramid fabric was used, produced by Teijin Aramid™ (Arnhem, The Netherlands). The aramid fabric’s technical characteristics are shown in Table 1. It is important to point out that plain-woven fabrics are produced by the interlacement of two sets of yarn (warp yarn and weft yarn) in a one-up and one-down manner. This is one of the simplest weave patterns possible to make by looms [41,42]. These plain-woven fabrics are commonly used in the production of ballistic helmets.

**Figure 1:**
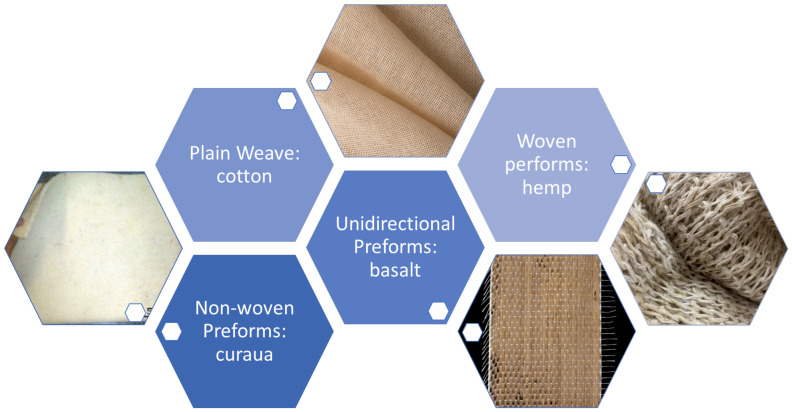
The most relevant textile preforms.

Curaua fiber was also used to reinforce the composites. It was in the form of a non-woven mat, Figure 1, produced by Pematec Triangel do Brasil. Table 2 presents a brief comparison of some natural fiber properties with application in textile production shown in Figure 1. Despite a large amount of research on polymeric matrix composites reinforced only with natural lignocellulosic fiber for ballistic applications, the possibly most promising use of these fibers in ballistic composites is in conjunction with synthetic fibers. Production cost and weight can be reduced by replacing part of the synthetic fibers without impairing ballistic performance. In addition to this, reducing the environmental impact resulting from the production of synthetic fibers and the disposal of ballistic composites reinforced only with them are great advantages of this novel possibility of observing [40,43].

**Figure 2:**
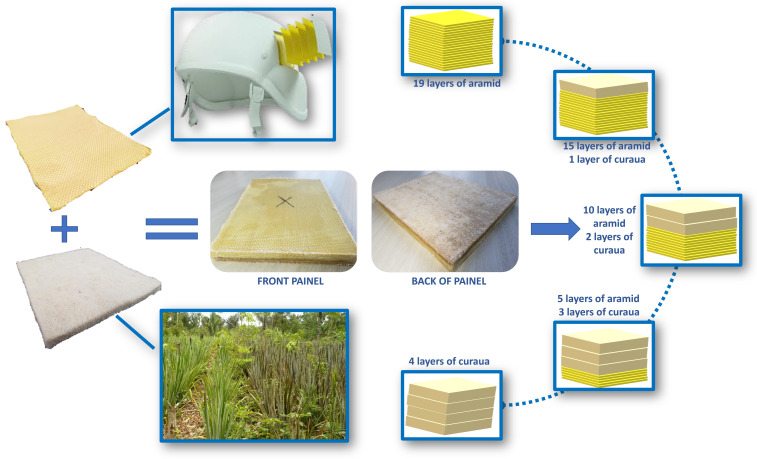
Diagram of the material used, aramid fabric and curaua non-woven mat, and three proposed configurations of hybrid laminated composites in the present work.

The Personnel Armor System for Ground Troops (PASGT) combat helmet, currently adopted by the Brazilian Army and others around the world, is manufactured with composite material of Polyvinyl butyral (PVB) matrix reinforced with aramid fabric. The laminate has about 20% m/m matrix and 19 layers of fabric. Based on this, five configurations of composites for ballistic helmets were proposed, as shown in Table 3, which shows the layers amounts of aramid fabric and curaua non-woven mat in each configuration. A diagram of the material used, aramid fabric and curaua non-woven mat, and three configurations of hybrid laminated composites proposed in the present work are presented in Figure 2.

In light of the difficulty of producing composites with a high level of reinforcement without the use of prepreg, as well as the use of natural fibers, the proposed configurations were idealized with up to 60 vol.% of reinforcement. Regarding the possibilities of matrix materials, epoxy resins were the materials that provide strength, durability, thermal properties, and chemical resistance to a composite, reducing the cost of composite application [46,47]. Furthermore, due to the ease of acquisition, storage and working of epoxy resin in contrast to the PVB-phenolic resin film. Additionally to its lower cost, the epoxy was used as a matrix, which, moreover being thermosetting, also has excellent mechanical properties. The epoxy resin has high specific strength, excellent adhesion, low weight, good dimensional stability and rigidity, and is widely used in various industries [48]. In summary, epoxy resin was chosen because it has high specific strength and hardness, high chemical resistance, good processability, is resistant to weathering, and relatively inexpensive.

### 2.2. Thermogravimetric Test

Thermal analyses by thermogravimetric (TGA), thermogravimetric derivative (DTG), and differential thermal analysis (DTA) were performed, based on the ASTM E1131-20 standard [49], for the epoxy matrix, aramid fabric, curaua non-woven mat and for the proposed hybrid laminated composites (Table 1), in order to determine the decomposition temperatures. The comminuted samples were placed in a platinum crucible and subjected, under a flow of 50 mL of nitrogen, to a heating cycle of 25–700 °C at a rate of 10 °C/min. For this, Shimadzu DTG-60H equipment (São Paulo, Brazil) was used.

### 2.3. Thermomechanical Test

Thermomechanical analysis (TMA) is a technique in which a deformation of the sample under non-oscillating stress is monitored versus time or temperature while the temperature of the sample in a specified atmosphere is programmed. TMA was carried out using Shimadzu equipment (São Paulo, Brazil), model TMA-60. The samples were prepared in accordance with the ASTM E831 standard [50], placed on a quartz support, under a flow of 50 mL of nitrogen, with a temperature range of 30–180 °C, at a rate of 10 °C/min and a fixed compression load of 50 gf. From this analysis, measurements of expansion were extracted, determined by the coefficient of thermal expansion (CTE), the glass transition temperature (T_g_), and the compression modulus.

## 3. Results And Discussion

### 3.1. Thermogravimetric Analysis

**Figure 3:**
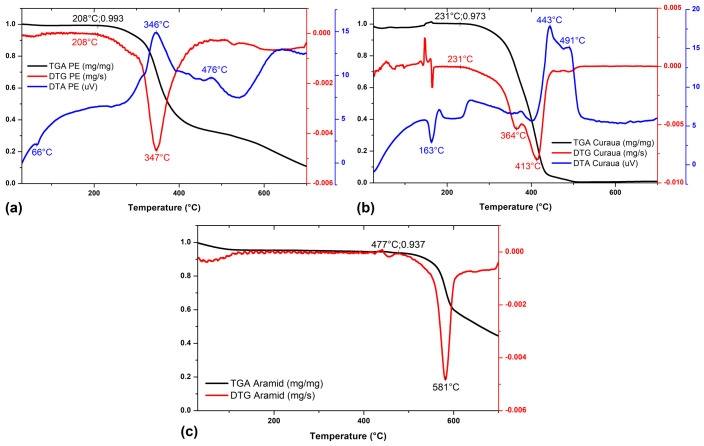
TGA (black line), DTG (red line) and DTA (blue line) curves of (**a**) epoxy system, used as matrix; (**b**) curaua mat and (**c**) aramid fabric, used as reinforcement in hybrid laminated composites.

Figure 3 shows TGA, DTG, and DTA curves of plain epoxy system, used as hybrid composite matrices, as well as aramid fabric and curaua non-woven mat, both used as reinforcement. One can see in Figure 3a that the epoxy thermal decomposition process starts at about 208 °C. Indicated by the beginning of the DTG curve peak, associated with an exothermic process. This process is represented by the DTG curve peak at the same temperature. DTG curve peak occurs at around 347 °C and corresponds to the material maximum thermal decomposition (MTD) rate. This phenomenon is represented in the TGA curve by a sharp drop in the mass fraction of the sample from 208 °C onward. It can be observed that, up to this temperature, sample mass loss is negligible, probably associated with the low humidity present in synthetic polymeric resins. It is important to point out that, in the DTA curve, at around 66 °C, an inflection point occurs, possibly corresponding to epoxy glass transition (T_g_).

Regarding the curaua non-woven mat, used as reinforcement in hybrid laminated composites, Figure 3b shows the thermal decomposition process of natural curaua fibers begins at around 231 °C. This is indicated by the beginning of the DTG curve peak, formed by two minor peaks that occur at about 364 °C and 413 °C. These secondary peaks correspond to the rate of maximum thermal decomposition of lignin and cellulose/hemicellulose, respectively [51]. The thermal decomposition of curaua fibers is represented in the TGA curve by a sharp drop in the mass fraction of the sample from 231 °C. It can be seen that, up to this temperature, sample mass loss is around 3%, probably associated with moisture present on the fiber’s surface. In the DTA curve, at around 163 °C, there is a peak associated with an endothermic process [1], corresponding to the elimination of absorbed or combined water between room temperature and 163 °C [52,53].

**Figure 4:**
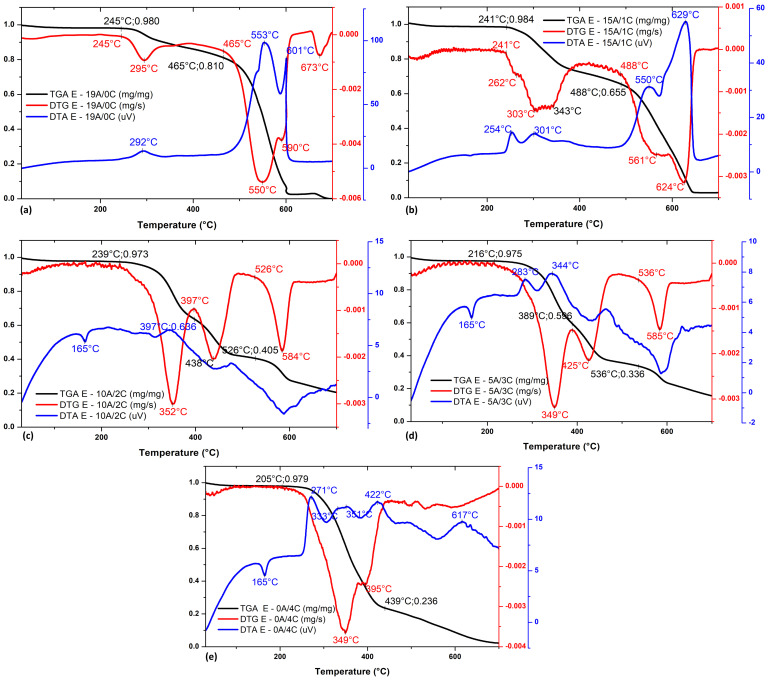
TGA (black line), DTG (headline) and DTA (blue line) curves of (**a**) E−19A/0C, (**b**) E−15A/1C, (**c**) E−10A/2C, (**d**) E−5A/3C, (**e**) E−0A/4C hybrid laminated composites.

One can also see in Figure 3c the TGA and DTG curves of aramid fabric used as reinforcement in hybrid laminated composites. It can be seen that the thermal decomposition process of aramid starts at about 477 °C. It is attributed to the beginning of the DTG curve peak, which occurs at around 581 °C and corresponds to the material MTD rate. It is represented in the TGA curve by a sharp drop in the mass fraction of the sample from 477 °C onward. It can be seen that, up to this temperature, sample mass loss is around 6%, probably associated with moisture present on the fiber’s surface. TGA, DTG, and DTA curves of E-19A/0C, E-15A/1C, E-10A/2C, E-5A/3C, and E-0A/4C composite are shown in Figure 4.

It can be observed in Figure 4a that E-19A/0C composite thermal decomposition process starts at about 245 °C. This is indicated at the beginning of the DTG curve peak at around 295 °C, associated with an exothermic process related to the DTA peak at 292 °C. Based on Figure 3a, it is important to note that the initial temperature of epoxy decomposition in the composite is considerably delayed compared to plain epoxy (Figure 3a). Epoxy thermal decomposition is represented in the TGA curve by a drop in the mass fraction of the sample from 245 °C. Up to this temperature, sample mass loss is around 2%, associated with moisture in the composite. At about 465 °C, aramid thermal decomposition begins to occur (Figure 4a) indicated by the onset of a new peak on the DTG curve at around 550 °C, associated with an exothermic process, which is represented by DTA curve peak at 553 °C. This phenomenon is represented in the TGA curve by a sharp drop in sample mass fraction from 465 °C onward. It is also important to highlight that, both in DTG and DTA curves, the peak corresponding to aramid thermal decomposition is formed by two secondary peaks at around 550 °C and 590 °C. The secondary peak at around 590 °C possibly corresponds to the thermal decomposition of aramid shielded by epoxy residues, which could increase activation energy to start the process.

Regarding the E-15A/1C composite, Figure 4b shows that its thermal decomposition process starts at about 241 °C, indicated by the onset of the first DTG curve peak, and is associated with an exothermic process. This process is represented by two exothermic DTA curve peaks at around 254 °C and 301 °C, which correspond to epoxy and cellulose/hemicellulose thermal decomposition, respectively. As their temperatures MTD rates are close, a single peak appears in the DTG curve between 200 °C and 400 °C, formed by two secondary peaks at around 262 °C and 303–343 °C. The result is a single accentuated drop on the TGA curve between 200 °C and 400 °C. At about 488 °C, aramid thermal decomposition begins to occur. This is indicated by the onset of a new peak on the DTG curve, formed by two minor peaks at around 561 °C and 624 °C. This phenomenon is represented in the TGA curve by a sharp drop in sample mass fraction from 488 °C onward. Up to this temperature, sample mass loss is around 35%, associated with almost all epoxy and curaua present in the composite. The onset of epoxy decomposition in E-15A/1C composite is considerably delayed compared to plain epoxy (Figure 3a). This is possibly due to the shielding of the aramid fabric.

**Figure 5:**
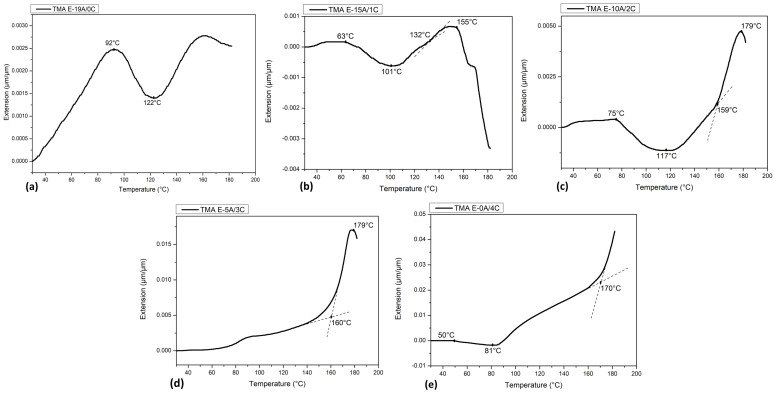
(**a**) E−19A/0C, (**b**) E−15A/1C, (**c**) E−10A/2C, (**d**) E−5A/3C and (**e**) E−0A/4C composite thermal extension and compressive stress-strain curves.

The E-10A/2C (Figure 4c) and E-5A/3C (Figure 4d) composites’ thermal decomposition process starts at around 239 °C, and 216 °C, respectively. This is indicated by the onset of the DTG curve’s first peak at 352 °C for the E-10A/2C composite and 349 °C for the E-5A/3C composite, both associated with the exothermic process. The E-5A/3C composite process is represented by two exothermic peaks of the DTA curve at around 283 °C and 344 °C, which correspond, respectively, to epoxy and cellulose/hemicellulose thermal decomposition. It is important to point out that, as their temperatures and MTD rate are close, a single peak appears in the DTG curve between 300 °C and 400 °C for both thermal decompositions. The result is a single sharp drop, in the TGA curve, between 300 °C and 400 °C. It can be seen that, up to these temperatures, sample mass loss is around 3%, associated with moisture release. At about 397 °C for E-10A/2C composite and 389 °C for E-5A/3C composite, thermal decomposition of lignin begins to occur. This is indicated by the onset of a new peak on the DTG curve at around 438 °C for E-10A/2C composite and 425 °C for E-5A/3C composite. This phenomenon is represented in the TGA curve by a sharp drop in sample mass fraction from 397 °C for E-10A/2C composite and 389 °C for E-5A/3C composite. Up to these temperatures, sample mass loss is around 35% for the first one and 40% for the second one, probably associated with epoxy and curaua in composites. It is important to highlight that this peak was evident in the DTG curve for these composites due to the higher amount of natural fiber in them compared to E-15A/1C composite.

At about 526 °C for E-10A/2C composite, and 536 °C for E-5A/3C composite aramid thermal decomposition begins to occur. This is indicated by the onset of a new peak on the DTG curve at around 584 °C for the first and 585 °C for the second. This phenomenon is represented in the TGA curve by a drop in sample mass fraction from 526 °C for E-10A/2C composite and 526 °C for E-5A/3C composite. Up to these temperatures, sample mass loss is around 60% for the first and 66% for the second, probably associated with almost all of the epoxy and curaua present in composites. Based on Figure 3a, it can be seen that the onset of epoxy decomposition in E-10A/2C and E-5A/3C composites was delayed in relation to plain epoxy, although this phenomenon was much more significant for the first one. This is possibly due to the higher amount of aramid in the E-10A/2C composite. Furthermore, based on Figure 3b,c, it can be observed that the beginning of curaua decomposition in composites did not suffer significant variation, but, for aramid, it was considerably delayed. It may be due to a shielding effect produced by natural fiber and its remains after burning. Furthermore, it can be observed that both for E-10A/2C, Figure 4c, and E-5A/3C, Figure 4d, in DTA curve, at about 165 °C, there was also a peak associated with an endothermic process, related curaua non-woven mat.

It can be seen that E-0A/4C (Figure 4e) composite thermal decomposition process starts at about 205 °C. This is indicated by the beginning of the DTG curve peak, associated with an exothermic process. This process is represented by three exothermic peaks of the DTA curve at around 271 °C, 333–351 °C, and 422 °C, corresponding to epoxy thermal decomposition, cellulose/hemicellulose, and lignin, respectively. Based on Figure 3a, it is important to highlight that the beginning of epoxy decomposition in composite did not change compared to that of plain epoxy. Likewise, based on Figure 3b, it can be observed that the beginning of curaua decomposition in composite also did not suffer significant variation. Up to 205 °C sample mass loss is around 2%, associated with moisture present in composite. As temperatures of epoxy and cellulose/hemicellulose MTD rates are close, a single peak appears in the DTG curve at around 349 °C, representing the decomposition of these elements. This also happened for lignin, but it is still possible to identify a secondary peak at around 395 °C. The result is a single drop in the TGA curve. Furthermore, it can be observed in the DTA curve at about 165 °C that there is also a peak associated with an endothermic process, as shown in Figure 3b.

It is suggested that the hybridization of aramid fabric with curaua non-woven mat in epoxy matrix composite was positive in terms of composite thermal behavior. In relation to E-19A/0C composite, there is no significant variation in temperature at the beginning of the decomposition process of E-15A/1C and E-10A/2C composites, around 240 °C. Furthermore, for E-10A/2C composite, the start of the aramid decomposition process was considerably delayed compared to E-19A/0C and E-15A/1C composites. It may be due to a shielding effect produced by natural fiber and its remains after burning.

### 3.2. Thermomechanical Analysis

**Figure 6:**
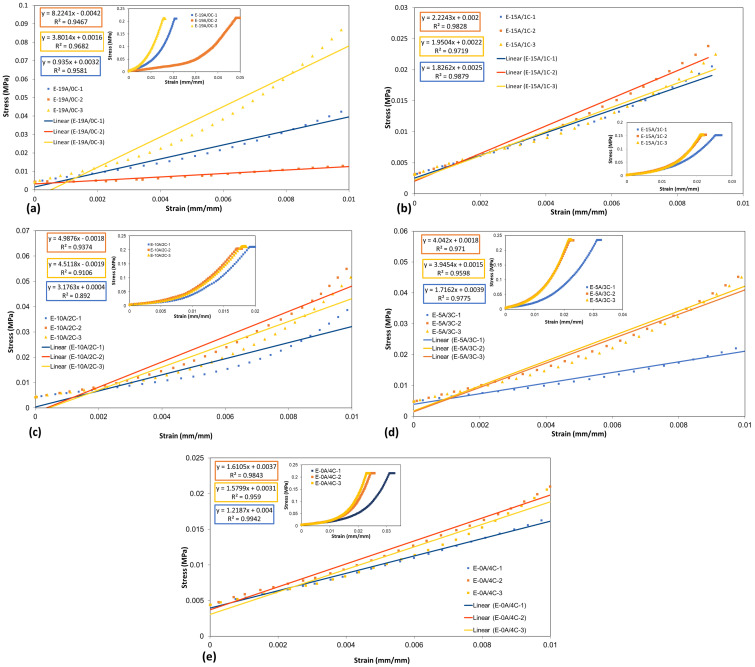
(**a**) E−19A/0C, (**b**) E−15A/1C, (**c**) E−10A/2C, (**d**) E−5A/3C and (**e**) E−0A/4C composite thermal extension and compressive stress-strain curves.

Figure 5 presents the measurements of expansion, determined by the coefficient of thermal expansion (CTE), as well as the glass transition temperature (T_g_) of E-19A/0C, E-15A/1C, E-10A/2C, E-5A/3C, and E-0A/4C composites, obtained with TMA. In addition, the compression modulus is shown in Figure 6.

The E-19A/0C composite, Figure 5a, presented contraction between 92 °C and 122 °C, probably due to the reduction of composite moisture. The T_g_ is determined by the point where a change in the coefficient of expansion occurs, in this case next to 160 °C [54]. This type of measurement is generally applied to materials that have relatively large coefficients of thermal expansion. Based on the compressive stress-strain curves from the sample of TMA, shown in Figure 6a, the Young’s modulus (YM) was calculated for deformations smaller than 0.01 mm/mm (1%) (YM${}_{1\%}$) and 0.005 mm/mm (0.5%) (YM${}_{0.5\%}$). The values obtained for the thermal decomposition temperature up to 5% of all materials, as well as the Young’s modulus results, are briefly presented in Table 4.

The YM${}_{1\%}$ was found to be 4.3 ± 3.7 GPa, with high variation, around 85%, and YM${}_{0.5\%}$ was obtained in the order of 2.7 ± 2.1 GPa, with smaller variation, around 79%. One can observe that there is a high variation among the samples, even considering only 19 layers of aramid fabric.

Regarding E-15A/1C composite, Figure 5b, it can be seen that from about 63 °C to 101 °C, the composite contracted due to reduced moisture. This is corroborated by the TGA curve of E-15A/1C composite, shown in Figure 4b. From about 101 °C, the sample gradually expands to 132 °C when the expansion rate increases. This is possibly due to epoxy T_g_, which increases the molecules’ mobility. At about 155 °C, the composite undergoes intense contraction. This is reinforced by the endothermic event at about 163 °C shown in curaua fiber DTA curve (Figure 1b). Figure 6b presented that the YM${}_{1\%}$ was found to be 2.0 ± 0.2 GPa, with variation around 10%, and YM${}_{0.5\%}$ was obtained in the order of 1.6 ± 0.2 GPa, maintaining the observed variation. It is noteworthy, therefore, that there is a decrease in the YM of up to 53.5%.

As can be seen in Figure 5c, E-10A/2C composite also contracted, probably due to reduction in moisture from about 75 °C to 117 °C. This is supported by the TGA curve of E-10A/2C composite shown in Figure 4c. From about 117 °C, E-10A/2C composite expands gradually up to 159 °C, when expansion rate increases considerably. Once again, this is possibly due to epoxy T_g_ [54]. At about 179 °C, the E-10A/2C composite undergoes intense contraction, as also observed for the E-15A/1C composite. This is reinforced by the endothermic event at around 165 °C shown on composite DTA curve (Figure 4b). It is important to highlight that, compared to E-15A/1C composite, glass transition and this event increased significantly. Equal values were also obtained for the E-5A/3C composite, as shown in Figure 4d.

This means that, T_g_ around 160 °C for E-19A/0C composite, was similar to the observed for E-10A/2C and E-5A/3C composites. It is important to emphasize that this temperature is significantly higher when compared to temperatures of use for ballistic protection. In addition, the glass transition temperatures of epoxy and this event in E-10A/2C and E-5A/3C composites are equal and higher than in E-15A/1C composite. For E-10A/2C composite, Figure 6c, YM${}_{1\%}$ was found to be 4.2 ± 0.9 GPa, with variation around 22%, and YM${}_{0.5\%}$ was obtained in the order of 2.2 ± 0.6 GPa, with almost the same observed variation (26%). Thus indicating a reduction of only 2.3% in relation to the E-19A/0C, however, with a smaller standard deviation. For E-5A/3C composite, YM${}_{1\%}$ was found to be 3.2 ± 1.3 GPa, with variation around 41%, and YM${}_{0.5\%}$ was obtained in the order of 2.3 ± 0.9 GPa, with 37% of variation. Thus indicating a reduction of only 25.6% in relation to the E-19A/0C, once again with a smaller standard deviation.

Figure 5e shows the thermal expansion curve of E-0A/4C composite. It can be seen that from about 50 °C to 81 °C, the sample contracted, probably due to the reduction of moisture in the composite. This is corroborated by the TGA curve of E-0A/4C composite, shown in Figure 4e. From about 81 °C, the sample gradually expands to about 170 °C, when the expansion rate increases considerably. This is possibly due to epoxy T_g_. Unlike the TMA curves of E-15A/1C, E-10A/2C, and E-5A/3C composites, that of E-0A/4C does not show an intense drop up to 180 °C, test limit temperature. However, the DTA curve for this composite (Figure 4e) indicates an endothermic event at about 165 °C. It may mean that the temperature corresponding to the onset of this contraction was greater than 180 °C and not necessarily the non-occurrence of this phenomenon. For E-0A/4C composite, YM${}_{1\%}$ was found to be 1.5 ± 0.2 GPa, with variation around 15%, and YM${}_{0.5\%}$ was obtained in the order of 1.1 ± 0.1 GPa, with almost the same observed variation (11%). Thus indicating a large reduction of only 65.1% in relation to the E-19A/0C, however, with a smaller standard deviation. It is important to highlight the trend towards greater thermal expansion and a reduction in compressive strength as the curaua non-woven mat content increases.

**Figure 7:**
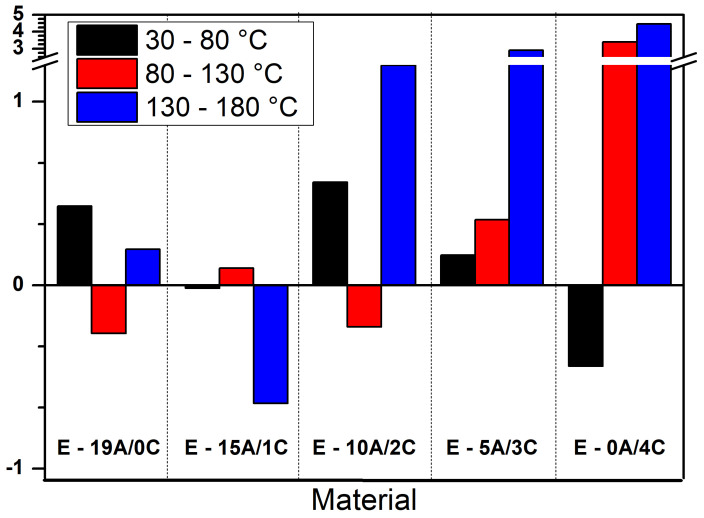
Plot of coefficient of thermal expansion for laminated composites with epoxy matrix.

The TMA expansion mode provides data regarding linear thermal expansion coefficients and the transitions detected by changing these coefficients, i.e., changes in the slope of the expansion curves; thus, Figure 7 shows the thermal coefficients obtained for the curves presented in Figure 5, with observation of slope changes occurring in three main temperature ranges, namely: 30–80 °C, 80–130 °C, and 130–180 °C.

The value of expansion coefficient determined on 30–80 °C interval increases in order: E-0A/4C < E-15A/1C < E-5A/3C < E-19A/0C < E-10A/2C, while on 80–130 °C interval increases in order: E -19A/0C < E-10A<2C < E-15A/1C < E-5A/3C < E-0A/4C. On 130–150 °C temperature interval, an increase of the expansion coefficient value by the replacement of aramid fabric layers with curaua non-woven mat layers was observed.

In the first interval, analyzed from RT to 80 °C, the most common alteration was expansion, which may be associated with the release of residual compressive stress as justified by Yamaguchi et al. [55]. For the second interval, between temperatures from 80 °C to 130 °C, both expansion and contraction phenomena were observed. In this study, it was not possible to relate the negative coefficient of thermal expansion presented by some samples with secondary polymerization, as suggested by Vaidyanathan et al. [56].

Additionally, like the others, the third and last interval, measured between temperatures from 130 °C to 180 °C, showed an expansion with a sudden increase in the coefficient of thermal expansion. It seems valid to suggest that this expansion is related to the T_g_ also advocated by Vaidyanathan et al. [56] and Sideridou et al. [57]. The coefficients of linear thermal expansion were found to be 6, 15, and 23 times greater than the reference (E-19A/0C composite), considering the E-10A/2C, E-5A/3C, and E-0A/4C composites samples, respectively.

## 4. Summary and Conclusions

In this paper, the thermal properties, based on thermogravimetric analysis (TGA), derivative thermogravimetric (DTG), differential thermal analysis (DTA), and thermomechanical analysis (TMA), of hybrid laminated curaua non-woven mat and aramid fabric reinforced epoxy composites were investigated, aiming at future applications in ballistic helmets. It is suggested that the hybridization of aramid fabric with curaua non-woven mat in epoxy matrix composite was beneficial in terms of composite thermal behavior.

In relation to the TGA technique, the epoxy system, curaua non-woven mat, and aramid fabric presented only one drop in the TGA curve. E-19A/0C composite, there was no significant variation in temperature at the beginning of the decomposition process of E-15A/1C and E-10A/2C composites. For E-10A/2C composite, the start of the aramid decomposition process was considerably delayed compared to E-19A/0C and E-15A/1C composites. It may be due to a shielding effect produced by natural fiber and its remains after burning. Despite that, in the DTA curve of E-10A/2C, E-5A/3C, and E-0A/4C composite, at around 165 °C, there was a peak associated with an endothermic process, which is related to curaua non-woven mat.

Regarding the TMA technique, a high variation in Young’s modulus of E-19A/0C composite samples was observed. Furthermore, a decrease in this property of up to 53% for E-15A/1C, 2% for E-10A/2C, 25% for E-5A/3C, and 65% for E-0A/4C, with, in all conditions, obtaining standard deviation values lower than those obtained by reference (E-19A/0C composite). The only sample that underwent just expansions was E-5A/3C; the others showed expansion and contraction behavior. In the third analyzed temperature interval, related to the glass transition temperature of the proposed materials, there was, in general, an expansive behavior, with the coefficient of linear thermal expansion 6, 15, and 23 times greater than the reference, considering the E-10A/2C, E-5A/3C and E-0A/4C composites samples, respectively.

## Figures and Tables

**Table 1:** Technical characteristics of the Twaron^®^ yarn from which the synthetic woven fabric used was manufactured.

Model	Linear Density	Twaron Type	Style	Set (per 10 cm) Warp and Welf	Area Density (g/cm^2^)	Thickness (mm)
T750	3360 f2000	1000	Plain	67	460	0.65

**Table 2:** Comparison of properties of some natural fiber with application in textile production [44,45].

Fibers	Cellulose (wt.%)	Hemicellulose (wt.%)	Lignin (wt.%)	Density (kg/m^3^)	Young’s Modulus (GPa)
Curaua	73.6	9.9	7.5	1100	20–36
Cotton	88–96.5	5.7	-	15,000	5.5–13.0
Basalt	-	-	-	2630	79.3–110
Hemp	70.2–74.4	17.9–22.4	3.7–5.7	1070	35

**Table 3:** Composite configurations based on the number of reinforcing layers [40].

Composite Configuration	Number of Layers	
	Aramid Fabric (A)	Curaua Non-Woven Mat (C)
Plain Epoxy (PE)	0	0
E-19A/0C	19	0
E-15A/1C	15	1
E-10A/2C	10	2
E-5A/3C	5	3
E-0A/4C	0	4

**Table 4:** Summary of results obtained.

Material	TGA_5%_ (°C)	Young’s Modulus (GPa)	
		YM_0.5%_	YM_1%_
E-19A/0C	291	2.7 ± 2.1	4.3 ± 3.7
E-15A/1C	278	1.6 ± 0.2	2.0 ± 0.2
E-10A/2C	290	2.2 ± 0.6	4.2 ± 0.9
E-5A/3C	285	2.3 ± 0.9	3.2 ± 1.3
E-0A/4C	274	1.1 ± 0.1	1.5 ± 0.2
Aramid	313	-	-
Curaua	307	-	-
Epoxy	281	-	-

## Data Availability

The data presented in this study are available on request from the corresponding author.
